# Within-item strategy switching in arithmetic: a comparative study in children

**DOI:** 10.3389/fpsyg.2013.00924

**Published:** 2013-12-10

**Authors:** Eléonore Ardiale, Patrick Lemaire

**Affiliations:** Centre National de la Recherche Scientifique, Aix-Marseille UniversitéMarseille, France

**Keywords:** strategies, arithmetic, switch costs, executive functions, cognitive development

## Abstract

The present study aimed at determining whether (1) children were able to interrupt a strategy execution to switch and choose another better strategy, and (2) their ability to switch strategy within-item improved with age. Third, fifth, and seventh graders performed a computational estimation task in which they had to provide the better estimates to two-digit addition problems (e.g., 32 + 54) while using the rounding-down (e.g., 30 + 50) or the rounding-up strategy (e.g., 40 + 60). After having executing the cued strategy (e.g., 30 + 50) during 1,000 ms, participants were given the opportunity to switch to another better strategy (e.g., 40 + 60) or to repeat the same strategy (e.g., 30 + 50). The results showed that children switched strategies within items, and were able to switch more often when the addition problems were cued with the poorer strategy (e.g., 40 + 60 for 32 + 54) than when cued with the better strategy (e.g., 30 + 50). As they grew up, children based their decisions to switch strategies more often on whether the 1,000-ms strategy execution concerned the better strategy or strategy difficulty (i.e., the rounding-up strategy). These findings have important implications to further understand mechanisms underlying within-item strategy switching as well as strategic variations in children.

## INTRODUCTION

In many domains, children like adults use several strategies to perform cognitive tasks (e.g., arithmetic, Siegler and Lemaire, 1997; Lemaire et al., 2004; Caviola et al., 2012; reasoning, Hartley and Anderson, 1983; Zimmerman, 2000; memory, Dunlosky and Hertzog, 2001; Schlagmüller and Schneider, 2002; language, Kail et al., 2012). A strategy is defined as “a procedure or a set of procedures for achieving a higher level goal or task” (Lemaire and Reder, 1999, p. 365). For example, when children want to grab a candy jar on a high shelf, they have several ways to achieve this goal: (a) standing on tiptoe, (b) taking an object (e.g., spatula) to pull the jar, (c) climbing on a stool, or (d) asking someone taller. According to changing environments (e.g., school, home, store), children like adults must determine which strategy is the most appropriate for each situation. Thus, choosing a strategy among several available strategies involves to use the same strategy as used previously for a similar context (e.g., home), and switching strategy in a different context (e.g., from home to school) in order to be more adaptive to each situation. For example, Campbell and Austin (2002) showed that adults adapted their strategy choices to the problem and situation contexts when solving addition problems. In other words, adults preferred to directly retrieve in memory the answer to small addition problems (i.e., 2 + 3) with a short response time deadline (i.e., 750 ms), and they used more frequently procedural strategies to solve large addition problems (i.e., 8 + 7) for longer response time deadline (i.e., 2,500 ms). Unfortunately, sometimes the chosen strategy is not the most effective one. In these cases, we wish we had an opportunity to make a better choice. The present work contributes to our understanding of strategy selection when children are given the opportunity to interrupt execution of an on-going strategy in order to choose a better strategy.

Computational models of strategies [e.g., Lovett and Anderson’s (1996) ACT-R model; Lovett and Schunn’s (1999) RCCL model; Payne et al.’s (1993) adaptive decision maker model; Rieskamp and Otto’s (2006) SSL model; and Siegler and Araya’s (2005) SCADS^*^ model] account for how children select strategies and for strategic development by assuming that children select strategies on a trial-by-trial basis. These models also assume that based on past experience, children select more and more frequently the better strategy on each problem. So, when children have to solve a new problem, they assess problem features, they activate strategies available to solve the present problem, select the most strongly associated strategy with the problem to be solved or with a related problem, execute the selected strategy, and store strategy performance relative to the problem features. Associative mechanisms are the key component of these models’ assumptions and have been proven sufficient to account for most findings on strategy choices and execution such as the effects of problem difficulty or strategy characteristics.

Previous empirical and theoretical works on strategies and strategic development had one limitation. Computational models and empirical works assume that children and adults choose and execute strategies on a trial-by-trial basis. Thus, the possibility that once engaged in strategy execution, the on-going chosen and executed strategy may be revised, has been neglected. In other words, because children have been found to select the better strategy most of the times, previous works have not considered the fact that strategy selection could be an iterative process, helping participants to continually assess the chosen and executed strategy’s efficiency, so as to interrupt their initially selected strategy during its execution and to switch to a better strategy. Therefore, the present study aimed at determining whether children are able to revise their initial strategy execution, and, if yes, how this changes with children’s age.

Recently, Ardiale and Lemaire (2012, 2013) provided some empirical evidence that in a particular context, adults interrupted a strategy mid-execution to switch and select a better strategy than the initially executed one, on the same problem. The authors defined a 1,000-ms execution time as sufficient (1) to be engaged in the cued strategy execution without having completely executing the rounding-up or the rounding-down strategy, and (2) to assess problem features and deciding to switch strategy or not in order to choose the better strategy. They also showed that such within-item strategy switching involved cognitive switch costs. In other words, participants’ performance (i.e., solution latencies and accuracy) was better when the same strategy was used after a 1,000-ms strategy execution than when switching to an alternative strategy. According to previous work on task and strategy switching (e.g., Allport and Wylie, 2000; Luwel et al., 2009; Lemaire and Lecacheur, 2010; Meiran, 2010), the authors interpreted these within-item strategy switch costs as the result of the involvement of executive functions.

Following on Ardiale and Lemaire’s (2012) experiments, we asked third, fifth, and seventh graders to find approximate sums of two-digit addition problems like 34 + 57, while either rounding-up (e.g., doing 40 + 70) or rounding-down (e.g., doing 30 + 50) both operands to their nearest decades. These two rounding strategies have been shown to be spontaneously used by children in forth and sixth grade while performing a computational estimation task (e.g., Lemaire et al., 2000; Lemaire and Lecacheur, 2002). Children started to execute a cued strategy (e.g., the rounding-down cued for 34 + 57) during 1,000 ms. Then, they were given the opportunity to choose to repeat the same strategy (i.e., the rounding-down strategy) or to switch to the alternative strategy (i.e., the rounding-up strategy) in order to end up executing the poorer or better strategy to solve the current problem (i.e., 34 + 57). In the current study, we manipulated the problem type (i.e., homogeneous problems vs. heterogeneous problems), the cued strategy (i.e., the better vs. the poorer rounding strategy) and the rounding strategies (i.e., the rounding-down vs. the rounding-up strategy). Our procedure allowed us to test several predictions. Developing children should improve on two levels. First, increased executive functions in children would allow children to interrupt an on-going strategy mid-execution and to switch strategies within item more often as they grow up. In other words, mean percentages of strategy switches would increase with age. Second, as assumed by strategy selection models, older children would have stronger problem–strategy associations, and thus would increasingly switch strategies to choose the better strategy. So, they would switch strategies more often when (1) it would be easier to determine the better strategy, that is for homogeneous problems (i.e., 21 + 34 or 79 + 86) comparative to heterogeneous problems (i.e., 24 + 37 or 71 + 89), and (2) the 1,000-ms cued strategy was the poorer strategy than when it was the better strategy. Thus, we tested age-related differences in within-item strategy switching while assuming that with age, children should have more cognitive resources to switch strategies more frequently and more adaptively. As rounding-up involves to increment and actively maintain decade digits, this strategy is harder or more resource-consuming than the rounding-down strategy. In other words, we expected that with age children would base their decision to switch strategies more often on whether the cued strategy is the poorer strategy rather than on the difficulty of the 1,000-ms executed strategy.

## MATERIALS AND METHODS

### PARTICIPANTS

Forty third graders (23 females; mean age = 8.7 years; age range = 7.1–10.0), 40 fifth graders (20 females; mean age = 10.7 years; age range = 9.1–12.1), and 42 seventh graders (24 females; mean age = 12.5 years; age range = 12.1–13.1) participated in this experiment. Children were recruited in two different public schools from Aix-en-Provence and Marseille (France).

### STIMULI

The stimuli were 100 addition problems presented in standard form (i.e., 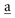 + 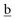) with the operands 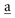 and 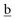 being two-digit numbers. The first manipulation of this experiment was the problem type. Twenty homogeneous and 80 heterogeneous addition problems were created. Homogeneous addition problems were used as control and were made so that participants, especially young children, would be able to easily choose the appropriate strategy. Half homogeneous addition problems had unit digits of both operands smaller than 5 (e.g., 34 + 52) and were better estimated with the rounding-down strategy (i.e., homogeneous, rounding-down problems). The other homogeneous problems had unit digits of both operands larger than 5 (e.g., 37 + 58) and were better estimated with the rounding-up strategy (i.e., homogeneous rounding-up problems). In heterogeneous problems, the unit digit of one operand was larger than 5 and that of the other operand was smaller than 5. Half of heterogeneous problems were rounding-down, meaning that they were better solved with the rounding-down strategy, and the other heterogeneous problems were rounding-up problems (i.e., better solved with the rounding-up strategy).

The better strategy on a given problem could be objectively determined by comparing the percent deviation between correct and estimated sum for each type of strategy calculated as |[(estimated sum - correct sum)/correct sum] × 100|. For example, percent deviations are 16.7 and 11.1% for 23 + 49 when estimated with the rounding-down and rounding-up strategy, respectively. Thus, rounding-up was the better strategy for this problem, whereas rounding-down was the better strategy for 32 + 46.

The second manipulation in this experiment was the cued strategy on each problem. Half the problems were cued with the better strategy (i.e., rounding-down problems cued with the rounding-down strategy), and the other problems were cued with the poorer strategy (i.e., rounding-up problems cued with the rounding-down strategy). The rounding-down strategy yielded the better estimates on half the problems (i.e., 31 + 42 or 32 + 46), and rounding-up was the better strategy on the other problems (i.e., 38 + 47 or 23 + 49). Size of correct sums and differences between correct sums and sums estimated with the rounding strategy (the rounding-down or the rounding-up strategy) were matched for the cued strategy (better or poorer strategy).

Problems were divided into two blocks of 50 addition problems each. Homogeneous and heterogeneous addition problems were equally distributed across the first and the second blocks. For each type of problem, mean correct sum and difference between correct sum and sum estimated with the cued strategy (better or poorer strategy) were equal between blocks (see stimuli characteristics’ in **Table 1**).

**Table 1:** Stimuli characteristics.

		Homogeneous problems		Heterogeneous problems	
		RD problems	RU problems	RD problems	RU problems
Mean percent deviation between correct sums and the better estimate (range)	Block 1	3.0 (2.3–3.7)	3.2 (2.2–5.3)	8.4 (4.5–11.8)	8.6 (4.9–12.9)
	Block 2	2.8 (2.1–3.2)	2.7 (2.0–3.7)	8.2 (5.1–11.4)	8.2 (4.9–12.7)
Mean percent deviation between correct sums and poorer estimate (range)	Block 1	11.7 (7.9–17.6)	11.9 (7.8–19.4)	11.9 (11.1–12.8)	12.0 (9.8–13.4)
	Block 2	11.3 (7.6–15.4)	11.2 (7.8–15.5)	12.2 (11.9–12.9)	11.7 (11.1–12.4)
Mean correct sum (range)	Block 1	134.0 (133–135)	131.8 (127–137)	107.9 (68–157)	106.1 (62–143)
	Block 2	133.7 (124–143)	138.8 (135–147)	108.4 (78–158)	109.1 (71–143)

Problems were selected so as to control for variables known to crucially influence arithmetic performance (see Campbell, 2005 or Geary, 1994 for reviews). We made sure that (a) no operands had 0 (e.g., 20 + 37) or 5 as unit digit (e.g., 25 + 37); (b) the decades were between 2 and 8; (c) digits were not repeated in the same decade or unit positions across operands (e.g., 23 + 27); (d) no digits were repeated within operands (e.g., 22 + 37); (e) no reverse order of operands were used (if 21 + 37 was used, 37 + 21 was not); (f) no tie problems (e.g., 21 + 21) were used; (g) the first operand was the largest in half the problems; (h) no operands had its closest decade equal to 0, 10, or 100.

### PROCEDURE

All children were tested individually in two sessions. Each session corresponded to 50 addition problems. Sessions were separated by 1 week on average (mean = 7 days; range = 1–15 days), and each session lasted approximately 30 min. At the beginning of each session, children were presented the computational estimation task. Computational estimation was explained as giving an approximate answer to an arithmetic problem that is as close as possible to the correct answer without actually calculating the correct answer. Children were told to use one of the two rounding strategies, the rounding-down or the rounding-up strategy. The rounding-down strategy was described as rounding both operands down to the nearest smaller decades, like when doing 30 + 50 to estimate 32 + 56. The rounding-up strategy was described as rounding both operands up to the nearest larger decades, like when doing 40 + 60 to estimate 32 + 56. Instructions emphasized that children should use no other strategy than the ones described above, and should do nothing more after calculating the product of rounded operands (i.e., adding or subtracting small amounts). Then, children were told to pay attention to the letter above the addition problems. The letter “B” (standing for “Bas” or “Down” in French) cued participants to solve the current problem with the rounding-down strategy, and the letter “H” (“Haut” or “Up” in French) prompted participants to use the rounding-up strategy. They were asked to calculate out loud using the cued strategy.

**FIGURE 1:**
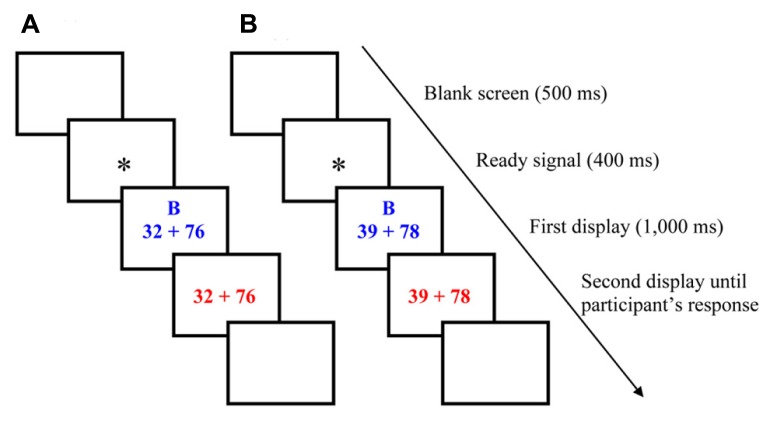
**Examples of trial progress: (A)** a heterogeneous rounding-down problem cued with the better, rounding-down, strategy, and **(B)** a homogeneous rounding-up problem cued with the poorer, rounding-down, strategy.

Stimuli were presented in 42-point Times New Roman font in the center of a 14-inch computer screen controlled by a DELL Latitude C540 laptop. The experiment was controlled by the E-Prime software. The program generated the displays and recorded latencies to the nearest millisecond. The symbol “+” and the numbers were separated by spaces equal to the width of one character. Each trial began with a 500-ms blank screen, followed by a 400-ms ready signal (“*”) appearing in the center of the screen. The addition problem and the cue letter above were displayed in blue during 1,000 ms. Then, the same addition problem was displayed alone (without the cue letter) in red until participant’s response (see **Figure 1**). Children were asked to start executing the cued strategy as soon as the problem and the cue appeared in blue on the computer screen. Children were told that, when the color of the problem changed from blue to red, they could change strategies if they judged that the current, cued strategy was not the better strategy (i.e., the strategy that provides the estimate closest to the correct sum). Again, children were asked to calculate out loud after the problem color changed in order to control whether participants had switched strategies or not. Each trial ended when participants gave an estimate.

Following many previous studies using the same type of computational estimation task (e.g., Lemaire and Lecacheur, 2010), a timer started when the color of the problem changed from blue to red and ended when the experimenter pressed the mouse button, which happened as soon as possible after participants finished giving their response orally. All participants received the same random order of homogeneous and heterogeneous problems.

Children received 20 training problems (respecting the same constraints but different from experimental problems) before each session to familiarize themselves with the apparatus, procedure (rounding strategies and problem display), and estimation task. After the two sessions, children completed an independent paper-and-pencil test in order to assess their arithmetic fluency. The arithmetic test consisted of one page of six lines of 10 addition problems. The stimuli were vertically presented, and had two operands either with two-digit numbers (e.g., 92 + 52) or one operand with a two-digit number and the other operand with a one-digit number (e.g., 99 + 5). All participants were given 6 min to solve the addition problems as fast and accurately as possible. A total arithmetic score was calculated from the number of correct answers. Solution times were also recorded for participants who completely achieved all addition problems before the 6 min ended. The mean scores were 30.1, 43.8, and 40.7 for third, fifth, and seventh graders, respectively. The difference between fifth and seventh graders was not significant, *F* < 2; but the differences between third graders and either fifth or seventh graders were significant (*F*s > 17.8).

## RESULTS

Results are reported in three main parts. The first part examines age-related differences in the ability to switch strategies within items (i.e., percentages of strategy switches). The second determines whether in each age group children based their decision to switch strategies on similar information (e.g., whether the cued strategy was the better or the poorer, the harder or the easier strategy). The third part looks at age-related differences in strategy switch costs (i.e., RTs and percentages of deviation).

We removed from analyses trials on which children did not start to execute cued strategies before the 1,000 ms ended, or started to execute another than the cued strategy. These trials represented 14.8, 10.4, and 10.6% of trials in third, fifth, and seventh graders, respectively. In all results, unless otherwise noted, differences are significant to at least *p* < 0.05 (see **Table 2** for significant statistics effects).

**Table 2:** Statistics of significant effects for mean percentages of switches, solution latencies, and percentages of deviation.

Effects	df	MSE	*F*	η^2^_p_
**Mean percentages of switches**			
**Homogeneous problems**			
Cued strategy	1,119	222,45	397.9	0.77
Age × Cued strategy	2,119	17,554	31.4	0.35
**Heterogeneous problems**			
Cued strategy	1,119	107,836	131.2	0.52
Age × Cued strategy	2,119	21,685	26.4	0.31
Age × Strategy	2,119	5,373	5.8	0.09
**Solution latencies**			
Age	2,119	88,535,545	15.69	0.27
Trial	1,119	3,434,334	25.15	0.23
Strategy	1,119	4,634,260	6.64	0.07
Trial × Strategy	1,119	10,001,216	8.94	0.09
**Percentages of deviation**			
Age	2,119	12.23	3.44	0.06
Age × Trial × Strategy	2,119	3.51	17.4	0.18

### AGE-RELATED DIFFERENCES IN PERCENTAGES OF STRATEGY SWITCHES

**FIGURE 2:**
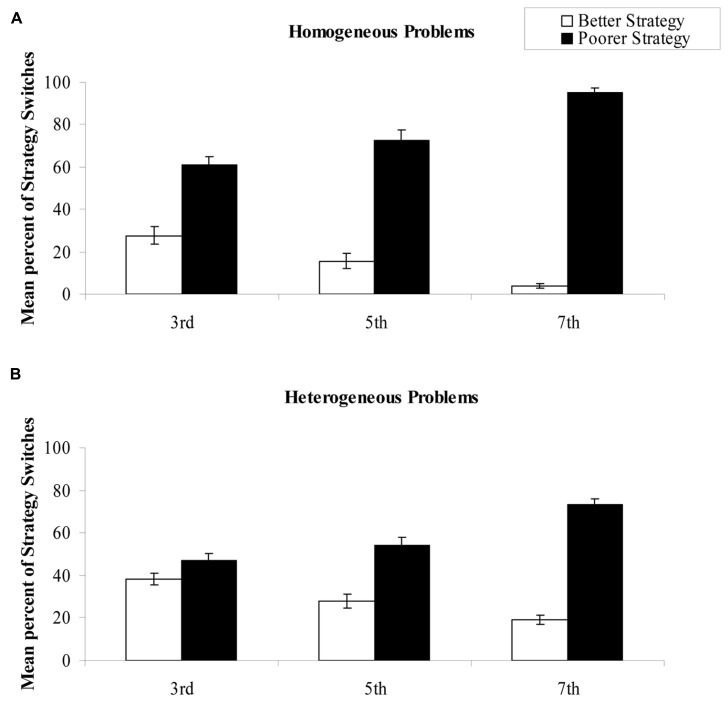
**Mean percentages of strategy switches for homogeneous (A)** and heterogeneous **(B)** problems in each Age × Cued Strategy condition.

We analyzed homogeneous (e.g., 31 + 42) and heterogeneous (e.g., 68 + 73) problems separately, with ANOVAs with 3 (age: third, fifth, and seventh graders) × 2 (cued strategy: better, poorer strategy) × 2 (strategy: rounding-down, rounding-up strategies)^1^ designs, with age as the only between-subjects factor (see mean values in **Figure 2**).

Regarding homogeneous problems (e.g., 21 + 34 or 38 + 57), children switched strategies within items more often when cued with the poorer (76.6%) than with the better strategy (15.7%). In order to better understand the Age × Cued Strategy interaction, we ran a one-way ANOVA with age as the only factor, on the difference between mean percentages of strategy switches for problems cued with the poorer and with the better strategies. This analysis revealed that the cued strategy effect (i.e., poor–better strategy) appeared to increase with age, *F*(2,119) = 31.3, MSE = 35,107, η^2^_p_ = 0.34, from 33.0 to 57.0%, respectively for third and fifth graders (*F* > 10.0), and to 91.2% for seventh graders (*F* > 21.0). This occurred because, as they grew older, children seemed to switch more and more often when cued with the poorer strategy, and less and less often when cued with the better strategy.

Regarding heterogeneous problems (e.g., 32 + 56 or 37 + 54), children switched strategies more often when cued with the poorer (58.1%) than with the better strategy (28.3%). To further analyze the presence of the Age × Cued strategy interaction, ANOVA involving the age factor as the only between-subjects factor was conducted on the difference between mean percentages of strategy switches when cued with the poorer and with the better strategies. The cued strategy effect appeared to increase with age, *F*(2,119) = 26.39, MSE = 821.6, η^2^_p_ = 0.31, from 8.9 to 25.9%, respectively for third and fifth graders (*F* > 7), and to 54.4% for seventh graders (*F* > 20.0). Again, this occurred because, as they grew older, children seemed to switch more and more often when cued with the poorer strategy, and less and less often when cued with the better strategy. Moreover, the presence of the Age × Strategy interaction suggested that fifth and seventh graders switched in 44.2 and 37.6% (*F* < 2.0) after executing the rounding-down strategy, and in 48.4 and 43.8% (*F* < 1) after executing the rounding-up strategy. However, third graders switched more often after executing the rounding-up (49.7%) than after the rounding-down strategy (35.4%). Complementary analyses on mean percentages of use of the rounding-down strategy as a function of age, *F*(2,119) = 6.09, MSE = 234.7, η^2^_p_ = 0.09, showed that third graders (58.0%) used the rounding-down strategy more frequently than older children (47.4 and 48.0%, respectively for fifth and seventh graders, *F* < 1).

### AGE-RELATED DIFFERENCES IN DETERMINANTS OF STRATEGY SWITCHES

Following our analyses in young and older adults (Ardiale and Lemaire, 2012), we ran a statistical analysis of the proportions of switches based on a generalized linear heterogeneous-effects (LME) model. We used the LME program (NLME package in the R system for statistical computing). Independent variables were considered in the analysis. Three were categorical factors: age (third, fifth, and seventh graders), strategy (rounding-down, rounding-up strategy), cued strategy (better, poorer strategy); and three factors were continuous: size of correct sums (e.g., 88 for 32 + 56), sum of unit digits (e.g., 8 for 32 + 56), and arithmetic fluency (i.e., scores at the French Kit test). The reference level of each factor was fifth graders for the age factor, the rounding-down strategy for the strategy, the better strategy for the cued strategy, and all of the continuous regressors (i.e., the last three variables mentioned above) were centered on their respective mean values (correct sum = 107; sum of unit digits = 10; and arithmetic fluency = 38.6). We also included possible interactions between age and other independent variables. We specified subjects as a random factor.

Results are displayed in **Table 3**. The estimate column corresponds to logit (switch proportion). We used the following inverse logit formula *y* = 1/[1 + (exp^-x^)] to transform the logit (switch proportion) value in percent of switches. The intercept estimate (-0.89) indicates average logit (switch proportion) when the categorical factors were at their reference level. This corresponds to an average of 29% 1/[1 + exp^-(-0.89)^] of switches for fifth graders, after partially executed the rounding-down strategy as the better strategy. Seventh graders also significantly switched strategies in 40% (1/[1 + exp^-(-0.42)^]. The mean percentage of strategy switches (a) was about 41% i.e., 1/[1 + exp^-(-0.89-0.37)^] after children partially executed the rounding-up strategy, (b) was about 77% i.e., 1/[1 + exp^-(-0.89+1.18)^] when they were cued with the poorer strategy, and (c) increased when the correct sum increased.

**Table 3:** Linear mixed effect model results.

	Estimate	Standard error	*z* Value	Pr(>|*z*|)
**Reference value: the fifth graders**				
Intercept	-0.89	0.13	-6.68	0.000***
Strategy	-0.37	0.09	-4.36	0.000***
Cued strategy	1.18	0.09	13.69	0.000***
Correct sum	0.00	0.00	2.11	0.035*
Sum of unit digits	0.02	0.02	0.74	0.458
Arithmetic fluency	-0.01	0.01	-0.89	0.371
**Age value: the third graders**			
Age	0.11	0.19	0.58	0.561
Age × Strategy	1.03	0.12	8.36	0.000***
Age × Cued strategy	-0.82	0.12	-6.64	0.000***
Age × Sum of unit digits	0.00	0.04	0.00	0.999
Age × Correct sum	0.00	0.00	-1.67	0.096
Age × Arithmetic fluency	0.01	0.01	0.40	0.689
**Age value: the seventh graders**			
Age	-0.42	0.18	-2.27	0.023*
Age × Strategy	0.09	0.13	0.74	0.460
Age × Cued strategy	1.27	0.13	9.94	0.000***
Age × Correct sum	0.00	0.00	-0.11	0.911
Age × Sum of unit digits	-0.03	0.04	-0.86	0.389
Age × Arithmetic fluency	0.01	0.01	0.49	0.628

Note: *p < 0.05, **p < 0.01, ***p < 0.001.

Interestingly, several interactions came out significant. The Age × Strategy interaction showed that the strategy was a significantly stronger determinant of strategy switches in third graders (slope = -037 + 1.03 = 0.66) than in fifth graders (slope = -0.37), or in seventh graders (slope = -0.37 + 0.09 = -0.28); the difference between fifth and seventh graders was not significant as the Age × Strategy interaction was not significant for seventh graders. However, seventh graders (slope = 1.18 + 1.27 = 2.45) based their decisions to switch strategies on whether the cued strategy was the better or the poorer strategy, more often than fifth graders (slope = 1.18) or than third graders (slope = 1.18 - 0.82 = 0.36).

### AGE-RELATED DIFFERENCES IN PERFORMANCE

We ran repeated-measures ANOVAs with 3 (age: third, fifth, and seventh graders) × 2 (trial: no switch, switch trials) × 2 (strategy: rounding-down, rounding-up strategies) design on mean solution times and percentages of deviation. Analyses revealed that third graders (15,844 ms) were slower than fifth or seventh graders (10,095 and 10,456 ms, respectively for fifth and seventh graders, *F* < 1). Children were faster when they repeated the strategy (11,593 ms) than when they switched strategies (12,670 ms) on the two problem displays. Children chose and executed a strategy slower after executing the rounding-up (12,453 ms) than the rounding-down strategy (11,810 ms). Moreover, the presence of the Trial × Strategy interaction revealed that (1) when children chose to repeat the rounding-up strategy (12,462 ms) they took more time than when repeating the rounding-down strategy (10,724 ms), *F*(1,119) = 11.17, MSE = 10,084,204, η^2^_p_ = 0.11, and (2) deciding to switch strategies was as fast after executing the rounding-down (12,896 ms) than the rounding-up strategy (12,443 ms), *F* < 2.

Third graders (10.2%) gave less accurate estimates than seventh graders (9.3%, *F*(1,119) = 17.4, MSE = 3.51, η^2^_p_ = 0.18), but third graders gave as accurate estimates as fifth graders (10.2%, *F* < 1), and likewise for fifth and seventh graders (*F* < 4). Finally, in order to better understand the presence of the Age × Trial × Strategy interaction, we analyzed the Trial × Strategy interaction in each age group. This interaction was present only in seventh graders, *F*(1,41) = 5.85, MSE = 2.71, η^2^_p_ = 0.13 and revealed that they were less accurate when repeating the rounding-up strategy (9.8%) than when switching from the rounding-up strategy to the rounding-down strategy (8.7%), *F*(1,41) = 12.77, MSE = 2.05, η^2^_p_ = 0.24.

## DISCUSSION

In the present study, we found that children were able to interrupt an on-going strategy execution to choose and execute a better strategy on a given problem. Moreover, children switched more often after having partially executed a poorer strategy. Interestingly, whether the cued strategy is the better or the poorer became a more important determiner of strategy switching with increasing age, and whether the strategy was the most difficult (vs. the easiest) was most important in younger children.

A surprising result was the lack of age-related differences in overall percentages of strategy switches. We expected that young children would have more difficulties inhibiting a partially executed strategy than older children. This would have resulted in less switching in younger children. Actually, this overall age-invariance in mean percentages of strategy switches masked important age-related differences regarding the type of items on which children switched strategies. Indeed, younger children switched inappropriately more often and appropriately less often than older children. The presence of the Age × Cued Strategy interaction showed increased effect of cued strategy with age. That is, younger children tended to switch more often than older children when cued with the better strategy and less often when cued the poorer strategy. In other words, younger children switched equally often when cued with the better or the poorer strategy, whereas fifth and seventh graders were able to switch strategy when cued with the poorer strategy and repeat the same strategy when cued with the better strategy.

It is possible that third graders switched strategies equally often when cued the better and the poorer strategy because they did not have enough time to properly assess problem features and/or strategy effectiveness on a given problem. Given that they are less experienced in arithmetic than older children, younger children may have weaker problem–strategy associations and maybe be are less skilled at executing basic arithmetic calculations. This lower arithmetic expertise made it more difficult for them to quickly determine the better strategy for each problem. One possible approach for future research to test this possibility is to manipulate duration of initial strategy execution. Participants could initially execute strategies for 1, 3, or 5 s before being given the opportunity to switch strategies. Such a design would offer the opportunity to test the prediction that with increasing duration of execution, younger children would switch more often appropriately as they would have more time to determine whether the selected strategy is the better or not.

Better strategy switching found here are consistent with previous research showing that children are more and more able to select the better strategy on each problem as they grow older (e.g., Siegler, 1988; Fuson, 1990; Lemaire and Siegler, 1995; Beishuizen et al., 1997; Blöte et al., 2001; Lucangeli et al., 2003; Luwel et al., 2005; Barrouillet et al., 2008; Kuhn and Pease, 2009; Lemaire and Calliès, 2009). Indeed, increased appropriate strategy switching combined with decreased inappropriate strategy switching with age led children in the present experiment to eventually use the better strategy more and more often as they grow older.

Note, however, that the present increase in the quality of strategy switching cannot be entirely explained by increased adaptive strategy selection that would result from increased arithmetic expertise in older children. Indeed, our analyses of determinants of strategy switching revealed that the most important determinant of strategy switching was the type of strategy (i.e., the easiest rounding-down vs. the hardest rounding-up strategy) for third graders and whether the cued strategy was the better or the poorer strategy in fifth and seventh graders. It seems that cognitive costs associated with the strategy to switch from was most important in younger children. That is, when engaged in the harder strategy execution, younger children were more reluctant to switch strategies even when they had to. This suggests that the cognitive costs associated with executing the harder strategy did not leave enough resources free in younger children for them to switch strategies. In contrast, older children, who are known to have more available processing resources, were able to base their decisions to switch strategies (or not) on whether the cued strategy was the better or the poorer. The fact that seventh graders were more able than fifth graders to do this systematically likely comes from seventh graders’ being more proficient in arithmetic than fifth graders.

An additional interesting unexpected finding in the present experiment was the lack of age-related differences in strategy switch costs (i.e., difference between performance when switching from a strategy to another one and when repeating the same strategy). Given increased efficiency of executive control processes underlying strategy switching with children’s age, we expected decreased strategy switch costs in older children. It is possible that strategy switching processes in within-item strategy switching do not completely overlap those underlying between-item strategy switching. Note, however, that the present set of data was not the most appropriate to test age-related decreased within-item strategy switch costs. Indeed, the present strategy switch costs, and their age-related changes, may be contaminated by the type of items on which children switched strategy. Recall that younger children often switched inappropriately and older children switched more often appropriately. This may have yielded decreased strategy switch costs in younger children, absorbing potential age-related differences. To validly compare within-item strategy switch costs, future research should first compare within-item strategy switch costs in children who switch on exactly the same types of items and equally often. This is possible if we use a cued paradigm (i.e., a cue indicates whether to switch strategy or not on each item after initially engaging in executing a strategy; see Ardiale and Lemaire, 2012, Experiment 2). With this approach, strategy switch costs would be unconfounded with proportions of switches and the type of problems on which children switch strategies. Consistent with this possibility, when focusing on only appropriate switching trials (i.e., when children switched when cued with the poorer strategy in the present study), we found that within-item strategy switch costs decreased from 2,161 to 996 ms in third to fifth graders and to 659 ms in seventh graders. This was found in spite of unequal frequencies of strategy switch costs across age groups. Data controlling for frequencies of switches and types of items on which children switch may show even larger age-related decreased strategy switch costs, a prediction that future research should test.

The present findings have important theoretical implications regarding our understanding of strategic development. One common assumptions of current computational models of strategy selection (e.g., ACT-R, Lovett and Anderson, 1996; RCCL, Lovett and Schunn, 1999; SCADS^*^, Siegler and Araya, 2005; SSL, Rieskamp and Otto, 2006; and the adaptive decision maker, Payne et al., 1993) is that children try to choose the better strategy on each problem. Moreover, all models but SCADS^*^ assume that, once they selected a given strategy, unless they cannot execute it accurately, execution runs to completion. The present data showed that children, like adults (Ardiale and Lemaire, 2012), are able to change strategies mid-execution if the selected strategy is not the best for that problem. Such findings are consistent with SCADS^*^ model of a strategy interruption mechanism. This mechanism was computationally plausible. The present data provide empirical evidence for its existence in children. Computational models of strategy selection other than SCADS^*^ (e.g., ACT-R, SSL, and RCCL) not yet assuming such an interruption mechanism could envision some extensions to include such a mechanism. One advantage of such extensions is that these models could articulate the computational constraints for how the strategy interruption mechanism works and how it changes with children’s age.

## Conflict of Interest Statement

The authors declare that the research was conducted in the absence of any commercial or financial relationships that could be construed as a potential conflict of interest.

## Footnotes

1 As there were fewer homogeneous than heterogeneous problems, the strategy factor was included in ANOVA design only for heterogeneous problems.
